# Susceptibility of *Neisseria gonorrhoeae* to Zoliflodacin and Quinolones in Hyogo Prefecture, Japan

**DOI:** 10.3390/pathogens14080831

**Published:** 2025-08-21

**Authors:** Takashi Yurube, Katsumi Shigemura, Yurino Kobayashi, Taishi Maeda, Nami Nishimura, Ayaka Yamada, Kotoko Kotani, Saki Horii, Hiroyuki Yoshida, Kayo Osawa

**Affiliations:** 1 Department of Orthopaedic Surgery, Kobe University Graduate School of Medicine, 7-5-1 Kusunoki-cho, Chuo-ku, Kobe 650-0017, Japan; takayuru-0215@umin.ac.jp; 2 Department of Urology, Teikyo University School of Medicine, 2-11-1 Kaga, Itabashi-Ku, Tokyo 173-8605, Japan; shigemura.katsumi.up@teikyo-u.ac.jp; 3 Department of Medical Technology, Faculty of Health Sciences, Kobe Tokiwa University, 2-6-2 Otani-cho, Nagata-ku, Kobe 653-0838, Japan; 212ma32@kobe-tokiwa.ac.jp (Y.K.); taishitaitai0228@gmail.com (T.M.); 192mb16@kobe-tokiwa.ac.jp (N.N.); 192mb36@kobe-tokiwa.ac.jp (A.Y.); 182ma25@kobe-tokiwa.ac.jp (K.K.); skmmb48@gmail.com (S.H.); 4 Hyogo Clinical Laboratory Corporation, 5-6-2, Aoyamanishi, Himeji 671-2224, Japan; hyoshida@hc-labo.co.jp

**Keywords:** *Neisseria gonorrhoeae*, zoliflodacin (ZFD), quinolones, susceptibility, DNA gyrase, topoisomerase IV

## Abstract

The DNA synthesis inhibitor zoliflodacin (ZFD) is expected to be effective against strains resistant to therapeutic agents for *Neisseria gonorrhoeae* infection. In addition to ZFD, we investigated the susceptibility of *N. gonorrhoeae* strains to ceftriaxone (CTRX), ciprofloxacin (CPFX), garenoxacin (GRNX), and sitafloxacin (STFX). Minimum inhibitory concentration values for ZFD and four other drugs were determined for 147 strains of *N. gonorrhoeae* isolated at medical institutions in Hyogo Prefecture, Japan, from 2015 to 2022. Amino acid alterations in *gyrA*, *gyrB*, *parC*, and *parE* were examined by polymerase chain reaction and sequencing analysis. Sequence type (ST) was determined for epidemiological analysis, and *N. gonorrhoeae* strains were classified. The non-susceptibility rate was not observed in CTRX. The lowest non-susceptibility rate was observed in ZFD (39.5%) compared to CPFX (80.3%), GRNX (83.7%), and STFX (65.3%) (all *p* < 0.0001). The most common amino acid alterations in *gyrA* and *parC* had non-susceptibility rates exceeding 80% to quinolones except ZFD, suggesting that these alterations may have influenced the resistance trend. STs were different between isolates in 2015 and those in 2020 and later. ZFD showed potent antimicrobial activity against *N. gonorrhoeae* strains that are highly resistant to quinolones. It may become a new option in the treatment of gonococcal infections.

## 1. Introduction

*Neisseria gonorrhoeae* causes the sexually transmitted infection gonorrhea, particularly among men who have sex with men, where asymptomatic carriers can exist in the throat and rectum. Symptoms of infection in men include urethritis, while in women, cervicitis or urethritis is noted. It can also be observed in both sexes in the external genitalia (throat, rectum, conjunctiva, and rarely throughout the body) [1,2]. According to the World Health Organization (WHO) data, an estimated 82.4 million new infections of *N. gonorrhoeae* are reported annually worldwide among adults aged 15–49 years [1]. Penicillin, azithromycin, and quinolones have been used as therapeutic agents, but resistant strains have been detected in many countries [3]. In Japan, intravenous ceftriaxone therapy is currently recommended, but strains with resistance genes have recently been isolated in several countries, including Japan, and worldwide, and single-dose, high-dose ceftriaxone or zoliflodacin (ZFD) regimens are coming out soon are needed [3]. Therefore, the development of an effective oral drug that is less burdensome to patients is underway.

ZFD is a novel antimicrobial agent, a spiropyrimidinetrione, with a distinct mechanism of action against bacterial Type II topoisomerases, the GyrB TOPRIM domain, which is different from fluoroquinolones, and it can be administered orally and is expected to be a treatment for gonococcal infections [4,5]. Like quinolones, it binds to and inhibits DNA gyrase and topoisomerase IV, the enzymes necessary for DNA synthesis. Compared to quinolones, which mainly target the DNA gyrase A subunit, ZFD mainly targets the B subunit and has a novel mechanism of action [4].

In this study, to confirm the efficacy of ZFD against *N. gonorrhoeae,* we planned to analyze antimicrobial susceptibility test trends and genes involved in resistance. We also confirmed the results of antimicrobial susceptibility testing and genetic analysis of ciprofloxacin (CPFX), garenoxacin (GRNX), and sitafloxacin (STFX), all of which are oral quinolones that inhibit DNA synthase in the same way as ZFD. These three drugs are quinolones of different generations, with CPFX being the second generation and GRNX and STFX the fourth generation. These were used together with ZFD to compare changes in minimum inhibitory concentration (MIC) due to differences in generation on the same strain. Moreover, we also performed antimicrobial susceptibility testing of ceftriaxone (CTRX), which is a recommended treatment for gonorrhea [1]. One of the main analytical goals of this study is to determine the annual trends in drug susceptibility based on molecular biological and genetic analyses. The general resistance mechanism of ZFD and quinolones is based on the quinolone resistance-determining region (QRDR), which is the gene encoding the A subunit of the DNA gyrase (*gyrA*), the gene encoding the B subunit (*gyrB*), the gene encoding the A subunit of topoisomerase IV (*parC*), and the gene encoding the B subunit (*parE*) [6,7,8,9]. To compare the results of antimicrobial susceptibility tests, we searched for amino acid alterations in these genes and confirmed what kind of relationship was involved. Furthermore, as an epidemiological analysis to determine the prevalence of *N. gonorrhoeae*, we determined the sequence type (ST) based on *N. gonorrhoeae* multiantigen sequence typing (NG-MAST). NG-MAST is one of the most well-known molecular typing methods. NG-MAST determines sequence types by combining sequences of two genes: *porB*, which encodes the porin B protein, a membrane porin channel protein, and *tbpB*, which encodes transferrin-binding protein B [10]. We also used it to identify the populations of *N. gonorrhoeae* strains.

## 2. Materials and Methods

### 2.1. Strains

A total of 147 *N. gonorrhoeae* strains were used (46 in 2015, 23 in 2020, 38 in 2021, and 40 in 2022), and they were collected from urethral and vaginal secretions in medical institutions in Hyogo Prefecture, Japan, in 2015, 2020, 2021, and 2022, and provided by the Hyogo Prefectural Clinical Laboratory.

### 2.2. Antimicrobial Susceptibility Testing

The MICs were determined for CTRX using the E-test method (bioMérieux Japan Ltd., Tokyo, Japan), for CPFX, GRNX, STFX, and ZFD using the agar plate dilution method, and for CTRX, CPFX, GRNX, and STFX using the clinical laboratory standards institute (CLSI) method [11]. For ZFD, the criteria of a previous study [12] were used as the reference: MIC ≥ 4 µg/mL was considered as resistant (R), 0.5–2 µg/mL as intermediate (I), and ≤ 0.25 µg/mL as susceptible (S) strains. Strains that were resistant (R) or intermediate (I) to any of the drugs were classified as non-susceptible (NS). The *N. gonorrhoeae* ATCC49226 strain was used as the control. In addition to a direct comparison of MICs against each antimicrobial agent, annual trends in the rate of non-susceptibility were also examined as evaluation items.

### 2.3. DNA Extraction, Detection of Amino Acid Alterations in QRDR Genes, and NG-MAST

Bacterial DNA from all *N. gonorrhoeae* strains tested for drug susceptibility was extracted by the boiling method (95 °C, 15 min) and centrifugation. Polymerase chain reaction (PCR) was performed using TaKaRa Ex Taq^®^ (Takara Bio, Shiga, Japan), and sequencing analysis for each PCR product was used to determine amino acid alterations in *gyrA*, *gyrB*, *parC*, and *parE* [6,7,8,9].

NG-MAST was conducted by PCR amplification and sequencing of two housekeeping genes (*porB* and *tbpB*) was performed to determine the ST [10].

### 2.4. Statistical Analysis

Data are presented as number of cases (%); analysis was performed using EZR (https://www.jichi.ac.jp/saitama-sct/SaitamaHP.files/statmed.html, accessed on 4 December 2024) [13] based on R (https://cran.r-project.org/, accessed on 4 December 2024), and *p* < 0.0500 was considered significantly different. The non-sensitivity rates of each drug were compared by the chi-square test or Fisher’s exact test using 2 × 2 tables, with >20% of expected frequencies being below 5. The effect of genetic alteration and epidemiological analysis on the non-sensitivity rate of each drug was examined by the chi-square test using 2 × 4 tables. In addition, annual trends were examined by the chi-square test using 2 × 4 tables and compared with the Bonferroni method adjustment (*p* < 0.0083 with significant difference) for the non-sensitivity rate among the annual strains.

## 3. Results

### 3.1. Detection of Antimicrobial Resistance Genes

The susceptibility results to CTRX, CPFX, GRNX, STFX, and ZFD of a total of 147 *N. gonorrhoeae* strains are shown in Table 1. The non-susceptibility rate was not observed in CTRX [0% (0/147 strains)], and it was the lowest rate among the five antibiotics (*p* < 0.0001). The non-susceptibility rates for CPFX, GRNX, STFX, and ZFD were 80.3% (118/147 strains), 83.7% (123/147 strains), 65.3% (96/147 strains), and 39.5% (58/147 strains), respectively. The non-susceptibility rate of STFX was significantly lower than those of CPFX (*p* = 0.0039) and GRNX (*p* = 0.0003). The non-susceptibility rate of ZFD was also significantly lower than those of CPFX (*p* < 0.0001), GRNX (*p* < 0.0001), and STFX (*p* < 0.0001). Thus, ZFD had the lowest non-susceptibility rate in the four antibiotics except CTRX.

### 3.2. Annual Trend of Non-Susceptible N. Gonorrhoeae Strains

**Figure 1:**
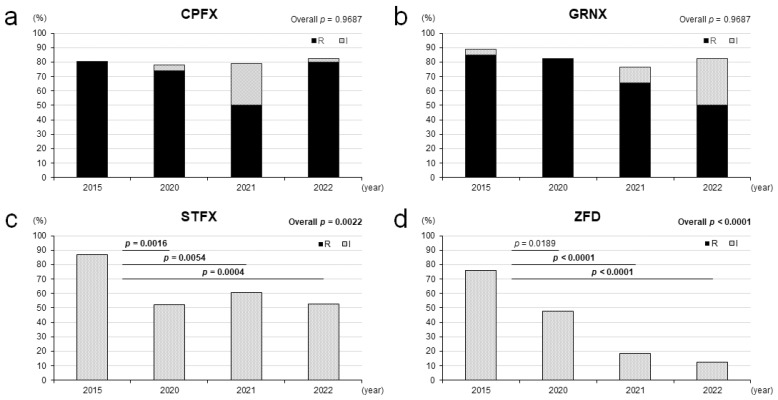
Annual trends of non-susceptibility rates of 147 *N. gonorrhoeae* strains to antibiotics. The results of the antimicrobial susceptibility testing of ciprofloxacin (CPFX) (**a**), garenoxacin (GRNX) (**b**), sitafloxacin (STFX) (**c**), and zoliflodacin (ZFD) (**d**) in *N. gonorrhoeae* strains show the rates of resistant (R) and intermediate (I) strains. All annual trends for the non-sensitivity rates were examined by the chi-square test, and each annual trend was compared among the 2015 and other years’ strains by the Bonferroni method adjustment (*p* < 0.0083 with a significant difference).

Figure 1 shows the annual trends of the non-susceptibility rates to CPFX, GRNX, STFX, and ZFD of the 147 *N. gonorrhoeae* strains isolated in 2015, 2020, 2021, and 2022. The 2015 strains were the initial strains from when we first started collecting and were used for comparison with strains from 2020 and later. The results showed that, of the four antibiotics examined, CPFX (*p* = 0.9731) and GRNX (*p* = 0.4867) both maintained non-susceptibility rates of around 80% from 2015 to 2022, compared to STFX (*p* = 0.0022) and ZFD (*p* < 0.0001). No significant differences were found in the year-by-year comparison between 2015 and 2020, 2021, or 2022 for non-susceptibility rates of strains for CPFX and GRNX (Figure 1a,b). The non-susceptibility rate of STFX was 87.0% in 2015, but it was 52.2–60.5% after 2020 (*p =* 0.0016 in 2020, *p* = 0.0054 in 2021, *p* = 0.0004 in 2022 versus 2015, after adjustment by the Bonferroni method as *p* < 0.0083 with a significant difference) (Figure 1c). On the other hand, ZFD showed a significant decrease in non-susceptibility rate over time from 76.1% in 2015 to 12.5% in 2022 (*p* = 0.0189 not reaching statistical significance in 2020, *p* < 0.0001 in 2021, *p* < 0.0001 in 2022 versus 2015, after adjustment by the Bonferroni method as *p* < 0.0083 with a significant difference) (Figure 1d).

### 3.3. Detection of Amino Acid Alterations in QRDR Genes

Table 2 shows the amino acid alterations in *gyrA*, *gyrB*, *parC*, and *parE* and the non-susceptibility of each mutant strain to CPFX, GRNX, STFX, and ZFD. In *gyrA*, the substitutions of aspartic acid at Ser91Phe (S91F) and position 95 including D95A, D95G, and D95N were frequently observed in 108 (73.5%) of 147 strains. Then, the non-susceptibility rates to CPFX, GRNX, and STFX of strains with S91F +D95A/G/N alterations in *gyrA* were 95.4%, 98.1%, and 75.9%, respectively. On the other hand, the strains with alterations at position 95 showed a low non-susceptibility rate of 39.8% to ZFD, which was statistically significant (*p* < 0.0001). The next most common alteration in *gyrA* was S91F (no difference in the non-susceptibility between groups, *p* = 0.2416), including D95G alteration (no difference in the non-susceptibility between groups, *p =* 0.4459). In *gyrB*, all strains had no alterations (a statistical difference in the non-susceptibility between groups, *p* < 0.0001). In *parC*, the serine substitution at position 87 was more common, with high non-susceptibility rates to CPFX, GRNX, and STFX at 96.5%, 100.0%, and 81.2%, respectively. However, the non-susceptibility rate to ZFD was low at 45.9%, even for the S87 alteration, and was statistically significant compared to other drugs (*p* < 0.0001), with S87R + S88P and S87N/R + E91G/K double alterations (*p* < 0.0001). In *parE*, the largest number of strains with no alteration was detected in 142 strains (a statistical difference in the non-susceptibility between groups, *p* < 0.0001), followed by D437N and P456S alterations in three and two strains, respectively.

### 3.4. NG-MAST

The results of the NG-MAST epidemiological analysis for *N. gonorrhoeae* strains are shown in Table 3. ST6800 and ST1407 appeared in strains in 2015, both presenting high non-susceptibility rates (78.6% to 100.0%) to all drugs (*p* = 0.1151 in ST6800 and *p* = 0.5508 in ST1407). The ST14149 strain, which has increased in number since 2020, has high non-susceptibility rates to CPFX (85.7%), GRNX (100.0%), and STFX (85.7%), but not to ZFD (28.6%) (*p* = 0.0105). Meanwhile, less than half of the strains in ST4207 were not susceptible to CPFX, GRNX, STFX, and ZFD, i.e., 12.5%, 0.0%, 25.0%, and 12.5%, respectively (*p* = 0.5153). Compared to the strains detected in 2015, the non-susceptibility rate to ZFD was 12.5–28.6% for the strain types that emerged in 2020 and later and decreased for all strains. Consequently, the results of this epidemiological analysis suggest that strains have changed from year to year, which may have influenced the decrease in the non-susceptibility rate of ZFD. On the other hand, there were also many not-typed and other strains in 2020 and later, with an increasing number of new strains that were not previously detected. Although quinolones are not recommended in the 2020 edition of the guidelines for the treatment of sexually transmitted diseases, there has been a trend toward the use of quinolones for some time, and it is undeniable that this may have had a small influence on the development of high quinolone resistance in the 2015 strains. Therefore, as a future perspective, although access to clinical data is not easy, we would like to proceed in future studies with comparisons of patient backgrounds, if possible, as well as the presence or absence of prior quinolone therapy, dosage, and duration of therapy.

## 4. Discussion

Antimicrobial susceptibility testing of 147 *N. gonorrhoeae* strains isolated in Hyogo Prefecture, Japan, in 2015, 2020, 2021, and 2022 showed that ZFD had the lowest non-susceptibility rate compared to CPFX, GRNX, and STFX except CTRX. Non-susceptibility of strains to CTRX was not detected. Genetic analysis further revealed that, among the amino acid alterations in the quinolone resistance region, substitutions at positions 91 and 95 were conspicuous in *gyrA* and at position 87 in *parC*. These findings suggest that the S91 and D95 alteration in *gyrA* and the S87 alteration in *parC* may have an impact on antimicrobial resistance to existing quinolones, except for ZFD. The S91F + D95A/G/N/Y alteration in *gyrA* and S87 H/I/CI/N/R alteration in *parC*, including the alterations found in the present study, have been identified in strains resistant to CPFX [12]. In this study, in *gyrB*, the strains with no alterations showed the lowest non-susceptibility to ZFD. In addition, *parE* had the highest number of strains with no alterations and showed the lowest non-susceptibility to ZFD, which inhibits the *gyrB* subunit, in contrast to quinolones, so alterations in *gyrB* may lead to increased ZFD resistance. It was known that amino acid alterations associated with the increased MIC of ZFD have been identified in D429N/A and K450N/T of *gyrB* in vitro [14], but none were detected in this study. *parE* alterations have been investigated less for their effect on quinolone resistance in *N. gonorrhoeae*. Similar to the results of the present study, no association between *parE* alterations and resistance has been confirmed in isolates from Nanjing, China [12]. As other mechanisms of resistance in *N. gonorrhoeae*, deletions of A in the promoter of the MtrCDE efflux pump (*mtrR*) and mutations in outer membrane proteins are also known, and the relationship with quinolone and ZFD resistance has not been reported [15,16]. Therefore, it is suggested that ZFD is an effective oral drug against *N. gonorrhoeae* compared to existing quinolones in the strains in this study, and no alteration related to resistance was specifically observed. If efficacy and safety are proven in future studies, ZFD may become a new therapeutic option in clinical practice.

Although further studies are needed, measures should be taken to address quinolone-resistant strains and CTRX-preservation therapy, and the history of rapid quinolone resistance should not be repeated. At the same time, the recent trend of increasing ZFD susceptibility suggests that the impact of recent quinolone sparing may have been a positive outcome. Future trends should be closely monitored [17,18,19,20].

ST determination by NG-MAST showed a change by generation, with ST6800 and ST1407 appearing in 2015, and ST14149 and ST4207 appearing after 2020. ST1407 is associated with reduced susceptibility and resistance to CTRX and fluoroquinolones globally, but recently new strains have been detected mainly in China [21]. In 2015, ST1407 showed non-susceptibility to quinolones and ZFD, but non-susceptibility to CTRX was not observed. Additionally, ST3435, which was identified as CTRX-resistant in 2015, was also not found (Supplemental Table S1) [22]. On the other hand, ST6800 was confirmed to have azithromycin resistance alongside ST1407 in Japan, exhibiting high non-susceptibility to ZFD and quinolones. Conversely, ST14149, which has been appearing since 2020, has a high non-susceptibility rate of more than 80% to quinolones except ZFD, and continued observation is needed for future fluctuations in resistance trends. On the other hand, ST4207 shows less than 50% non-susceptibility to quinolones and ZFD; azithromycin-resistant strains with ST4207 were identified in gonococcal isolates in Hyogo Prefecture in 2015–2019, though there have been no reports from other regions [21,23,24]. The latest WHO (2024) and CDC guidelines (2021) no longer recommend azithromycin dual therapy because of widespread macrolide resistance; so as mentioned above, single-dose, high-dose ceftriaxone or ZFD regimens are expected [25,26]. Continued epidemiological studies are needed to understand the trends in antimicrobial resistance in the treatment of these gonococcal infections.

In the Phase II trial conducted between 2014 and 2015, 96% of the patients administered 2 g of ZFD showed healing at the urogenital site. All rectal and pharyngeal infections were cured among those who received 2 g or 3 g of ZFD. In the absence of complications, the healing rate exceeded 95% [17,27]. It has been more than 10 years since the concept of antimicrobial resistance began to be more widely recognized, and many studies have been conducted. In recent years, many studies have been published on the increase in resistance to multidrug antimicrobial agents and the increase in resistant bacteria, but few drugs have a reduced resistance rate in gonococcal infections, which are included in the current WHO antimicrobial resistance alert threats. The significance of this study is great in that we were able to demonstrate this point.

The ST analysis was primarily an epidemiological study, and the annual trends in MICs for each drug were examined for clinical significance. Both studies consistently showed a decrease in ZFD non-susceptibility rates, and the results were highly reliable. Future development of clinical trials using ZFDs to treat conventional quinolone-resistant strains or patients who have failed conventional quinolone therapy are expected to compare the results with those of the CTRX treatment group. Further discussion is needed on whether 3 g of ZFD should be used in clinical trials.

There are few recent data on quinolone resistance rates after 2020, and it appears that more studies are being conducted on the percentage of multidrug-resistant *N. gonorrhoeae* and genetic analysis than on the evaluation of resistance rates worldwide. In Japan, a large-scale survey of the antimicrobial susceptibility testing of *N. gonorrhoeae* in 2005 revealed that 14.0% of 243 strains showed resistance to CPFX, and high-level CPFX-resistant strains were identified after 2001 [28].

Reports from the USA are very different from those from Japan, partly due to the difference in antimicrobial usage, with a resistance rate to CPFX of just over 30%. Although CPFX has not been included in the recommended treatment regime for gonorrhea since 2007, the proportion of CPFX-resistant *N. gonorrhoeae* strains increased from 31.2% to 35.4% (up 4.2%) from 2018 to 2019 among 1710 strains as part of the Gonococcal Isolate Surveillance Project (GISP) in the United States that collected 1710 *N. gonorrhoeae* strains in 2019 [15].

In Europe, there is much talk about azithromycin and ceftriaxone, and less about quinolones. In the 2009–2016 Gonorrhea Antimicrobial Surveillance Program (Euro-GASP), CPFX accounted for 51.7% of overall *N. gonorrhoeae* resistance [29]. In Asian countries, a 2022 Chinese study reported a very high resistance rate (97.6%) to CPFX [30]. Meanwhile, in *N. gonorrhoeae* strains isolated from Qatar from 2017 to 2020, the non-susceptibility rate to CPFX (*n* = 365) was 88.2% [31]. Our study found no significant change in the rate of insensitivity of CPFX, but STFX, as the same quinolone, showed the same decreasing trend over time as Qatar, and the new quinolone ZFD also showed a significant downward trend. The use of quinolones for this disease is clearly declining in Japan, and future changes in non-sensitivity rates should be monitored closely. In a report from China [32], the MICs of ZFD ranged from 0.002 µg/mL or less to 0.25 µg/mL. The overall MIC_50_ and MIC_90_ in 2018 were 0.06 µg/mL and 0.125 µg/mL, respectively, a two-fold increase from 2014, and the proportion of isolates with high ZFD MICs has been increasing year by year (*p* < 0.0001). These results differ from those of the present study, so evidence from Japan is still considered valuable.

Importantly, ZFD is theoretically effective against *gyrA* mutants, with the S91 and D95 alterations. In addition to this, the overall non-susceptibility rate increased, suggesting the need for future clinical trials in Japan. Like the mechanism of action of fluoroquinolones, the main target of ZFD in *N. gonorrhoeae* is bacterial Type II topoisomerases, the GyrB TOPRIM domain, whereas topoisomerase IV is a minor target [5,33]. Because the asymmetric targeting of gyrase and topoisomerase IV has promoted the evolution of fluoroquinolone resistance, it is important to understand the mechanisms underlying the differential targeting of ZFD in *N. gonorrhoeae.* Since ZFD is an effective oral medication, it is also important to consider the issue of the rapid emergence of resistance in hosts of gonococci in the pharynx [34].

The limitations of this study are that not all the strains analyzed were collected consecutively, there is no information other than the materials regarding the patients from whom the strains were obtained, and, therefore, clinical comparisons could not be made, and it is unclear whether this study shows all trends. In addition, interpreting the data requires caution due to a lack of continuity in the years of isolation of the strains. There was a discontinuity in the sampling for the years 2015, 2020, 2021, and 2022. Samples from 2016 to 2019 were also collected, and there were plans to conduct drug susceptibility tests; however, since the CLSI method uses the agar dilution method as the standard, it was difficult to perform the measurements easily, which is a factor that prevented accurate time trends from being reflected in the measurements for all years. One more point is that ZFD should be evaluated as a potential treatment for CTRX-resistant strains, but this study focused on quinolones as a comparison group and did not evaluate resistance to CTRX. Furthermore, no alterations in *gyrB* were found; however, it is necessary to consider conducting whole-genome sequencing that includes regions beyond codon 467. Lastly, this study did not include clinical data on the patients from whom the strains were isolated, such as prior antibiotic exposure, treatment history, or clinical outcomes. The lack of clinical data might affect the analysis of antimicrobial resistance. We focused on longitudinal findings of the analytical institution from a heterogenous population with *N. gonorrhoeae*, and this study limitation should be considered and overcome in our future study.

## 5. Conclusions

ZFD showed potent antimicrobial activity against 147 strains in Hyogo Prefecture in 2015, 2020, 2021, and 2022, including strains that were highly resistant to quinolones. Due to its different mechanism of action from conventional quinolones, it was observed that many strains that were resistant to quinolones were susceptible to ZFD. Furthermore, one of the important aspects of this study was the decrease in the non-susceptibility rate to ZFD in 2020 and later. If ZFD is approved based on its efficacy and safety, it may become a new option in the treatment of gonococcal infections and should be continuously investigated, including its resistance trends.

## Figures and Tables

**Table 1:** Susceptibility results of 147 *Neisseria gonorrhoeae* strains to antibiotics.

Antibiotic	No. of Strains (%)			Chi-Square Test (*p*)			
	NS			CTRX	CPFX	GRNX	GRNX
	R	I	S				
CTRX	0 (0.0)	0 (0.0)	147 (100.0)				
CPFX	105 (71.4)	13 (8.8)	29 (19.7)	<0.0001	−	−	−
GRNX	105 (71.4)	18 (12.2)	24 (16.3)	<0.0001	0.4481	−	−
STFX	0 (0.0)	96 (65.3)	51 (34.7)	<0.0001	0.0039	0.0003	−
ZFD	0 (0.0)	58 (39.5)	89 (60.5)	<0.0001	<0.0001	<0.0001	<0.0001

CPFX, ciprofloxacin; CTRX, ceftriaxone; GRNX, garenoxacin; STFX, sitafloxacin; ZFD, zoliflodacin; I, intermediate; NS, non-susceptible; R, resistant; S, susceptible. NS = R + I.

**Table 2:** Amino acid alterations in quinolone resistance genes in 147 strains of *N. gonorrhoeae*.

Amino Acid Alterations		No. of Non-Susceptibility Strains (%)				Total Strains
		CPFX	GRNX	STFX	ZFD	
*gyrA*	No alteration	7 (22.6)	9 (29.0)	8 (25.8)	10 (32.3)	31
	S91F + D95A/G/N	103 (95.4)	106 (98.1)	82 (75.9)	43 (39.8)	108
	S91F	6 (100.0)	6 (100.0)	5 (83.3)	4 (66.7)	6
	D95G	2 (100.0)	2 (100.0)	1 (50.0)	1 (50.0)	2
*gyrB*	No alteration	118 (80.3)	123 (83.7)	96 (65.3)	58 (39.5)	147
*parC*	No alteration	7 (22.6)	7 (22.6)	6 (19.4)	11 (35.5)	31
	S87H/I/N/R	82 (96.5)	85 (100.0)	69 (81.2)	39 (45.9)	85
	S87R + S88P	14 (93.3)	15 (100.0)	13 (86.7)	5 (33.3)	15
	S87N/R + E91G/K	10 (100.0)	9 (90.0)	5 (50.0)	2 (20.0)	10
	D86N	2 (100.0)	2 (100.0)	2 (100.0)	0 (0.0)	2
	E91G/K	2 (100.0)	2 (100.0)	1 (50.0)	0 (0.0)	2
	others	1 (50.0)	2 (100.0)	2 (100.0)	1 (50.0)	2
*parE*	No alteration	113 (79.6)	117 (82.4)	95 (66.9)	57 (40.1)	142
	D437N	3 (100.0)	3 (100.0)	0 (0.0)	0 (0.0)	3
	P456S	2 (100.0)	2 (100.0)	0 (0.0)	1 (50.0)	2

CPFX, ciprofloxacin; GRNX, garenoxacin; STFX, sitafloxacin; ZFD, zoliflodacin.

**Table 3:** Annual sequence type (ST) by NG-MAST of 147 strains of *N. gonorrhoeae*.

ST	Total Strains		No. of Non-Susceptibility Strains (%)			
	2015 Year	2020–2022 Years	CPFX	GRNX	STFX	ZFD
6800	14	0	11 (78.6)	14 (100.0)	14 (100.0)	12 (85.7)
1407	10	0	9 (90.0)	10 (100.0)	10 (100.0)	9 (90.0)
14,149	0	7	6 (85.7)	7 (100.0)	6 (85.7)	2 (28.6)
4207	0	8	1 (12.5)	0 (0.0)	2 (25.0)	1 (12.5)
NT	0	38	−	−	−	−
Others	22	48	−	−	−	−

PFX, ciprofloxacin; GRNX, garenoxacin; STFX, sitafloxacin; ZFD, zoliflodacin. NT, not typed.

## Data Availability

The data sets analyzed in the present study are available from the corresponding author on reasonable request.

## Supplementary Materials

The following supporting information can be downloaded at: https://www.mdpi.com/article/10.3390/pathogens14080831/s1. Table S1. The source of 147 strains of Neisseria gonorrhoeae.

## Abbreviations

The following abbreviations are used in this manuscript:

CLSI	clinical laboratory standards institute
CPFX	ciprofloxacin
CTRX	ceftriaxone
GRNX	garenoxacin
I	intermediate
MIC	minimum inhibitory concentration
NG-MAST	*N. gonorrhoeae* multiantigen sequence typing
NS	not susceptible
NT	not typed
PCR	polymerase chain reaction
QRDR	quinolone resistance-determining region
R	resistant
S	susceptible
ST	sequence type
STFX	sitafloxacin
WHO	World Health Organization
ZFD	zoliflodacin
