# Supplementary material for: Susceptibility of Neisseria gonorrhoeae to Zoliflodacin and Quinolones in Hyogo Prefecture, Japan
Source: Pathogens. 2025 Aug 21;14(8):831. doi: 10.3390/pathogens14080831 (PMC12389086; doi:10.3390/pathogens14080831)
Supplement: Supplementary file 1 [file pathogens-14-00831-s001.zip › pathogens-3811090-supplementary.pdf]

Supplemental Table S1. The source of 147 strains of *Neisseria gonorrhoeae*

Year	Strain No.	MIC(μg/mL)				E-test MIC(μg/mL)	Sequence Type			Amino acid alterations			
		CPFX	STFX	GRNX	ZFD	CTRX	*porB*	*tbpB*	ST	*gyrA*	*gyrB*	*parC*	*parE*
2015	1	0.004	0.002	0.016	0.25	<=0.002	10993	241	18833				
2015	2	0.031	0.125	8	1	0.094	4035	110	6800	S91F, D95G		S87R,A95P	
2015	3	4	0.5	2	1	0.023	8992	21	15406	S91F, D95G		S87R	
2015	4	2	0.125	2	1	0.047	908	110	1407	S91F, D95G		S87R	
2015	5	0.008	0.25	8	1	0.064	4035	110	6800	S91F, D95G		S87R	
2015	6	16	0.5	8	1	0.094	4035	110	6800	S91F, D95G		S87R	
2015	9	16	0.5	8	0.25	0.094	4035	110	6800			S87R	
2015	11	16	0.5	8	0.25	0.094	4035	110	6800	S91F, D95G		S87R	
2015	12	16	0.125	2	0.25	0.032	908	27	3588	S91F, D95G		S87R	
2015	13	16	0.5	4	0.5	0.125	1053	110	3702	S91F, D95G		S87R	
2015	14	0.008	0.5	4	0.5	0.047	908	110	1407	S91F, D95G		S87R	
2015	15	16	0.125	4	0.5	0.064	4035	110	6800	S91F, D95G		S87R	
2015	16	8	0.125	2	0.5	0.064	908	110	1407	S91F, D95G		S87R	
2015	17	1	0.125	2	1	0.047	908	110	1407	S91F, D95G		S87R	
2015	18	8	0.125	4	1	0.094	908	110	1407	S91F, D95G		S87R	
2015	19	16	0.125	8	0.5	0.032	4069	110	6780	S91F, D95G		S87R	
2015	20	8	0.125	4	0.5	0.064	4035	110	6800	S91F, D95G		S87R	
2015	22	16	0.25	4	0.5	0.064	908	10	7806	S91F, D95G		S87R,S88P	
2015	23	16	0.125	2	0.5	0.125	4035	110	1407	S91F, D95G		S87R	
2015	24	0.002	0.125	4	0.5	0.064	4035	110	6800	S91F, D95G		S87R	
2015	25	32	0.25	4	0.5	0.064	4035	110	6800	S91F, D95G		S87R	
2015	26	32	0.25	4	0.25	0.064	1053	110	3702	S91F, D95G		S87R	
2015	27	32	0.125	4	0.5	0.064	908	110	1407	S91F, D95G		S87R	
2015	28	8	0.125	4	0.5	0.047	908	110	1407	S91F, D95G		S87R	

2015	29	16	0.125	2	0.5	0.094	3003	110	4951	S91F, D95G		S87R	
2015	30	0.004	0.002	0.016	0.5	<=0.002	1704	27	4018				
2015	31	0.016	0.004	0.016	0.5	0.004	4028	241	6771				
2015	32	8	0.125	2	0.5	0.032	4033	110	6798	S91F, D95G		S87R,E91K	
2015	33	8	0.125	4	0.25	0.032	908	110	1407	S91F, D95G		S87R	
2015	34	0.008	0.004	0.016	0.5	0.004	4028	241	6771				
2015	35	8	0.125	8	0.5	0.047	4035	110	6800	S91F, D95G		S87R	
2015	36	16	0.125	8	0.25	0.094	8244	110	14236			S87R	
2015	37	16	0.125	4	0.5	0.023	4035	110	6800			S87R	
2015	38	8	0.125	2	0.25	0.064	9654	27	16614	S91F, D95G		S87R	
2015	40	8	0.25	2	2	0.047	4035	110	6800	S91F, D95G		S87R	
2015	41	8	0.125	4	2	0.094	908	110	1407	S91F		S87R	
2015	42	16	0.25	2	2	0.064	4035	110	6800	S91F, D95G		S87R	
2015	43	0.004	0.004	0.016	1	0.002	1704	27	4018				
2015	44	32	0.25	16	0.5	0.012	4429	10	7374			S87R,S88P	
2015	45	32	0.125	2	2	0.008	4429	10	7374	S91F		S87R,S88P	
2015	46	16	0.25	16	0.5	0.064	8951	110	15364	S91F, D95G		S87R	
2015	48	8	0.125	0.5	0.5	0.064	8244	110	14236	D95G		S87R	
2015	50	8	0.25	4	0.5	0.047	4035	110	6800			S87R	
2015	51	4	0.25	2	0.125	0.012	4429	10	7374			S87R,S88P	
2015	52	4	0.063	0.5	0.031	0.023	3498	27	14198	D95G		S87R	
2015	54	64	0.125	4	0.25	0.008	8257	4	14255	S91F, D95G		S87R,S88P	
2020	1	0.25	0.063	2	0.5	0.012	2481	2073	NT	S91F, D95G		S87R	
2020	2	0.008	0.004	0.031	0.5	0.003	5507	25	NT				
2020	3	4	0.063	4	0.25	0.012	954	756	NT	S91F, D95A		S87R	
2020	5	8	0.063	4	0.5	0.006	1838	21	3030	S91F, D95G			P456S
2020	7	32	0.125	16	0.5	0.064	7747	110	13354	S91F, D95G		S87R	

2020	8	16	0.25	8	0.5	0.023	4065	10	6778			S87R,S88P	
2020	9	16	0.063	4	0.25	0.19	8198	110	14181	S91F, D95G		S87R	
2020	13	0.063	0.125	8	0.25	0.012	8172	4	14149	S91F, D95G		S87R,S88P	
2020	15	0.016	0.002	0.031	0.25	0.004	1	25	NT				
2020	17	2	0.125	4	0.5	0.047	8246	110	14238	S91F, D95G		S87R	
2020	18	4	0.25	2	0.125	0.023	11	110	19669	S91F, D95G		S87R	
2020	19	4	0.125	8	0.25	0.047	8172	21	16759	S91F, D95G		S87R	
2020	20	4	0.063	1	0.5	0.023	954	21	NT	S91F, D95A		S87R	
2020	21	16	0.5	16	0.5	0.125	7861	21	NT	S91F, D95G		S87R	
2020	22	0.031	0.004	0.031	0.25	0.003	1808	25	16658				
2020	23	8	0.25	1	0.25	0.016	8	1307	NT				
2020	24	0.031	0.004	0.031	0.25	0.004	2161	25	16658				
2020	26	4	0.25	4	0.5	0.003	8172	4	14149	S91F, D95G		S87R,S88P	
2020	27	16	0.25	2	0.25	0.047	9654	2032	NT			S87R	
2020	29	4	0.063	1	0.125	0.004	17	2089	NT	S91F, D95G		S87R	
2020	30	16	0.25	4	0.5	0.032	7747	110	13354	S91F, D95G			
2020	31	4	0.063	2	0.5	0.032	1808	21	8394				
2020	32	8	0.125	4	0.125	0.023	16	60	NT	S91F, D95A		E91K	
2021	1	0.008	0.004	0.016	0.25	<=0.002	11803	25	NT				
2021	3	0.004	0.004	0.004	0.063	<=0.002	1808	25	4207				
2021	4	32	0.5	2	0.125	0.006	9385	110	16109	S91F, D95G		S87R	
2021	6	16	0.25	4	0.125	0.003	1132	21	13272	S91F, D95G		S87R	
2021	7	0.5	0.125	4	0.25	0.016	8176	110	16757	S91F, D95G		S87R	
2021	8	4	0.063	0.5	0.008	0.006	4054	21	14158	S91F, D95N		S87N	
2021	9	4	0.063	2	0.125	0.012	9838	953	16882	S91F, D95A		S87R	
2021	10	32	0.5	2	0.25	0.008	9743	2346	16718	S91F, D95N		D86N	
2021	11	16	0.5	2	0.25	0.012	1132	21	13272	S91F, D95G		S87R	

2021	12	32	0.125	2	0.125	0.004	9743	2346	16718	S91F, D95N		S87R	
2021	13	32	0.5	4	0.5	0.016	9739	3	16714	S91F, D95N		S87R	
2021	15	0.004	0.008	0.016	0.25	0.006	3361	25	14248				
2021	20	8	0.063	4	0.25	0.012	1808	21	8394	S91F, D95A		S87R	P456S
2021	22	4	0.063	4	0.5	<=0.002	954	21	NT	S91F, D95A		S87R	
2021	23	16	0.125	8	0.5	0.016	8172	4	14149	S91F, D95G		S87R	
2021	24	16	0.5	4	0.25	0.023	2566	21	4183	S91F, D95G		S87R	
2021	25	0.004	0.008	0.016	0.25	0.004	2193	27	3657				
2021	26	16	0.125	2	0.125	0.032	8173	110	14150	S91F, D95G		S87R	
2021	27	8	0.125	8	0.125	0.032	2526	60	NT	S91F, D95A		S87N	
2021	28	16	0.125	8	0.25	0.008	8172	4	14149	S91F, D95G		S87R	
2021	35	16	0.125	8	0.25	<=0.002	8172	4	14149	S91F, D95G		S87R	
2021	36	0.008	0.004	0.031	0.25	0.004	1808	25	4207				
2021	37	16	0.125	8	0.25	0.016	8172	4	14149	S91F, D95G		S87R	
2021	38	8	0.125	4	0.25	0.004	7984	21	13840	S91F, D95G		S87R	
2021	39	0.125	0.008	0.016	0.25	0.004	1808	25	4207				
2021	40	0.5	0.063	0.5	0.5	0.004	4028	241	6771	S91F, D95G			
2021	41	0.125	0.063	1	0.031	0.004	4054	21	14158	S91F, D95N		E91G	
2021	42	0.25	0.25	4	0.25	0.012	1132	21	13232	S91F, D95G		S87R	
2021	43	0.5	0.125	4	0.25	0.032	8172	25	NT	S91F, D95N		S88P	
2021	44	0.002	0.002	0.008	0.031	0.003	1808	241	16755				
2021	45	0.002	0.002	0.016	0.5	0.004	1808	25	4207				
2021	46	0.5	0.125	8	0.5	0.012	9739	60	NT	S91F, D95A		S87H	
2021	47	0.5	0.25	8	0.5	0.094	785	60	3611	S91F, D95A		S87N	
2021	50	0.5	0.125	8	0.25	0.003	621	2451	NT	S91F, D95G		S87R	
2021	51	0.25	0.5	2	0.25	0.006	4206	2346	NT	S91F, D95N		S87R	
2021	52	2	0.125	1	0.25	0.004	4054	21	14158	S91F, D95N		S87N	

2021	57	0.5	0.25	4	0.25	0.064	9654	110	NT	S91F, D95G		S87R	
2021	58	0.004	0.002	0.125	0.25	0.003	2193	27	3657				
2022	1	0.5	0.031	0.063	0.25	<=0.002	8357	241	NT	S91F, D95G			
2022	2	16	0.125	2	0.5	<=0.002	785	60	3611	S91F, D95A		S87N,E91K	
2022	3	8	0.25	1	0.25	<=0.002	9654	2451	NT	S91F, D95G		S87R	
2022	4	8	0.125	0.5	0.063	<=0.002	9053	21	NT	S91F, D95N		S87R,S88P	
2022	6	8	0.063	0.063	0.25	0.003	9739	60	NT	S91F, D95N		S87N,E91K	
2022	7	0.002	0.002	0.004	0.25	0.003	1808	25	4207			なし	
2022	9	8	0.125	0.5	0.25	0.004	2629	110	NT	S91F, D95G		S87R	
2022	10	16	0.125	1	0.25	0.004	4030	2346	NT	S91F, D95N		S87R,S88P	
2022	12	1	0.25	0.5	0.25	<=0.002	4054	21	14158	S91F, D95N		S87R,E91G	
2022	14	16	0.125	0.5	0.25	0.006	8919	2346	NT	S91F, D95N		S87R,S88P	
2022	16	0.002	0.125	0.008	0.25	0.008	1808	25	4207				
2022	17	16	0.25	0.5	0.5	0.008	9654	110	NT	S91F, D95G		S87R	
2022	18	0.002	0.004	0.5	0.25	0.008	19	18	19917				
2022	19	16	0.063	0.5	0.125	0.008	4054	21	14158	S91F, D95N		S87N,E91G	
2022	20	16	0.25	1	0.25	0.008	8198	1180	NT	S91F, D95G		S87R	
2022	23	2	0.063	2	0.25	0.012	8172	60	NT	S91F, D95N		S87N,E91K	
2022	24	64	0.25	2	0.25	0.012	8258	10	14256	S91F, D95A		S87R,S88P	
2022	26	16	0.5	2	0.25	0.012	8198	110	14181	S91F, D95G		S87R	
2022	27	2	0.125	2	0.25	0.012	11808	953	NT	S91F, D95A		D86N	
2022	28	0.002	0.008	0.5	0.25	0.016	2193	27	3657				
2022	30	32	0.5	2	0.25	0.016	4715	60	NT	S91F, D95A		S87N,E91K	
2022	31	8	0.063	1	0.008	0.016	9739	60	NT	S91F, D95A		S87N,E91K	
2022	32	8	0.031	0.5	0.063	0.016	4028	21	14157	S91F, D95A		S87R	
2022	33	16	0.125	2	0.125	0.016	785	60	3611	S91F, D95A		S87N,E91K	
2022	34	32	0.5	0.5	0.25	0.023	9071	110	15490	S91F, D95G		S87R	

2022	36	16	0.063	2	0.25	0.023	9708	4	16683	S91F, D95G		S87R,S88P	
2022	38	0.002	0.125	0.008	0.25	0.023	1808	25	4207				
2022	39	16	0.063	1	0.25	0.023	1417	2451	NT	S91F, D95G		S87R	
2022	40	64	0.25	1	0.063	0.023	8869	2346	NT	S91F, D95N		S87R,S88P	
2022	41	0.008	0.002	0.002	0.125	0.023	1808	25	4207				
2022	44	16	0.125	1	0.25	0.023	9654	2451	NT	S91F, D95G		S87R	
2022	45	16	0.063	2	0.25	0.03	8172	4	14149	S91F, D95G		S87R,S88P	
2022	47	16	0.25	1	0.25	0.032	9654	110	NT	S91F, D95G		S87R	
2022	49	0.063	0.063	0.004	0.5	0.032	3361	25	14248				
2022	50	8	0.125	0.5	0.5	0.032	8246	110	14238	S91F, D95G		S87R	
2022	52	16	0.016	0.25	0.016	0.047	1808	470	NT	S91F, D95N		S87I	D437N
2022	53	64	0.063	2	0.25	0.047	1733	470	23926	S91F, D95N		S87I	D437N
2022	54	8	0.063	0.5	0.5	0.064	9739	21	NT	S91F, D95N		S87R	
2022	56	64	0.063	2	0.125	0.094	1733	470	23926	S91F, D95N		S87I	D437N
2022	57	8	0.063	2	0.125	0.094	9739	60	NT	S91F, D95A		S87N,E91K	

*CTRX, ceftriaxone, CPFX, ciprofloxacin; GRNX, garenoxacin; STFX, sitafloxacin; ZFD, zoliflodacin.

ST: Sequence type, NT: Not typed
